# Advances in the Study of Heart Development and Disease Using Zebrafish

**DOI:** 10.3390/jcdd3020013

**Published:** 2016-04-09

**Authors:** Daniel R. Brown, Leigh Ann Samsa, Li Qian, Jiandong Liu

**Affiliations:** 1 Department of Pathology and Laboratory Medicine, University of North Carolina at Chapel Hill, Chapel Hill, NC 27599, USA; drbrown7@email.unc.edu (D.R.B.); li_qian@med.unc.edu (L.Q.); 2 Department of Cell Biology and Physiology; University of North Carolina at Chapel Hill, Chapel Hill, NC 27599, USA; lsamsa@email.unc.edu; 3 McAllister Heart Institute, University of North Carolina at Chapel Hill, Chapel Hill, NC 27599, USA

**Keywords:** zebrafish, congenital heart disease, cardiomyopathy, cardiac arrhythmia, development, translational medicine, drug screens

## Abstract

Animal models of cardiovascular disease are key players in the translational medicine pipeline used to define the conserved genetic and molecular basis of disease. Congenital heart diseases (CHDs) are the most common type of human birth defect and feature structural abnormalities that arise during cardiac development and maturation. The zebrafish, *Danio rerio*, is a valuable vertebrate model organism, offering advantages over traditional mammalian models. These advantages include the rapid, stereotyped and external development of transparent embryos produced in large numbers from inexpensively housed adults, vast capacity for genetic manipulation, and amenability to high-throughput screening. With the help of modern genetics and a sequenced genome, zebrafish have led to insights in cardiovascular diseases ranging from CHDs to arrhythmia and cardiomyopathy. Here, we discuss the utility of zebrafish as a model system and summarize zebrafish cardiac morphogenesis with emphasis on parallels to human heart diseases. Additionally, we discuss the specific tools and experimental platforms utilized in the zebrafish model including forward screens, functional characterization of candidate genes, and high throughput applications.

## 1. Introduction

Congenital heart diseases (CHDs) are the most common type of human birth defect and frequently exhibit structural abnormalities that arise from defective cardiac development and maturation [1,2,3]. These defects compromise cardiac output and lead to poor clinical outcomes. Though Mendelian genetics can explain some CHDs, differential penetrance of CHD phenotypes in affected families underscores the need for a better understanding of the cellular and molecular events of cardiac development [4]. The genetic networks that regulate vertebrate heart development are highly conserved across species enabling modeling of human heart developmental disorders in zebrafish [5,6]. Additionally, the zebrafish model system is relatively inexpensive and can be used in high-throughput compound screens to identify novel therapeutics for personalized medicine [7,8,9,10,11,12].

### Modeling Cardiovascular Development and Disease in Zebrafish

**Figure 1:**
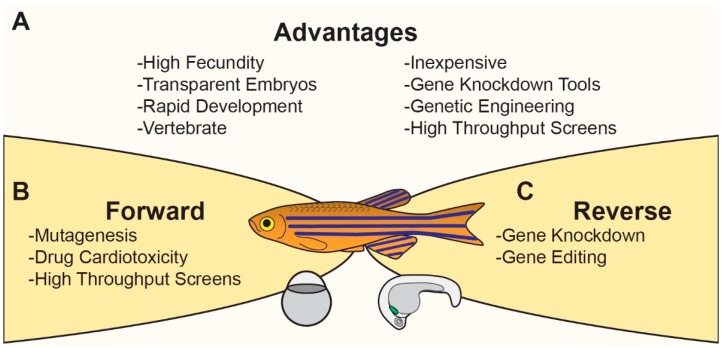
Zebrafish Model System. Schematic illustrating (**A**) the advantages of zebrafish as a model system, (**B**) forward genetic and (**C**) reverse genetic approaches to studying heart development and disease in zebrafish.

The zebrafish, *Danio rerio*, has emerged as a premier vertebrate model system for investigating the molecular basis of heart development and assessing therapeutic potential of small molecules [13,14,15]. Zebrafish have several key advantages over other vertebrate model systems that are inherent to their biology (Figure 1A) [16]. A single breeding pair produces hundreds of eggs weekly, facilitating genetic and statistical analysis. These externally fertilized eggs develop rapidly, and by 24 hours post fertilization (hpf), the embryonic heart has initiated cardiac contraction. Though the zebrafish heart has a simpler structure than the human counterpart (two chambers instead of four chambers), it possesses analogs of the major components of the human heart and utilizes similar cellular and molecular strategies to assemble the heart [5,17]. Due to the transparency of the embryos, the morphology and function of the developing hearts can be directly observed by light microscopy. This optical transparency can also be leveraged by the use of transgenic reporters in which cardiac cells are labeled with fluorescent markers [18,19,20,21]. Importantly, zebrafish embryogenesis does not require a functional cardiovascular system during the first week of life because the zebrafish embryo is small enough to meet oxygenation needs by diffusion [22,23,24,25,26]. This allows for examination of severe cardiovascular defects that usually cause embryonic lethality in other model organisms, such as mice. These advantages allow for robust forward and reverse genetic approaches to study the genetic and molecular basis of heart development and disease (Figure 1B,C).

In this review, we will provide an overview of zebrafish heart development, and discuss how zebrafish are leveraged to study cardiovascular development and disease.

## 2. Cardiovascular Development in Zebrafish

The heart is the first organ to form and function during vertebrate embryonic development. The key steps of heart development are conserved across vertebrates, and the gross morphological changes associated with cardiac morphogenesis have been well described in detail in previous reviews [5,15,27,28,29]. Additionally, we refer the interested reader to the online Zebrafish Atlas (http://zfatlas.psu.edu) for histological details of zebrafish development from embryo to adult. Histology methods are also readily available for zebrafish [30,31]. Below, we will overview zebrafish cardiac morphogenesis and disease phenotypes placing an emphasis on parallels to human heart disease.

### 2.1. Zebrafish Cardiac Morphogenesis

**Figure 2:**
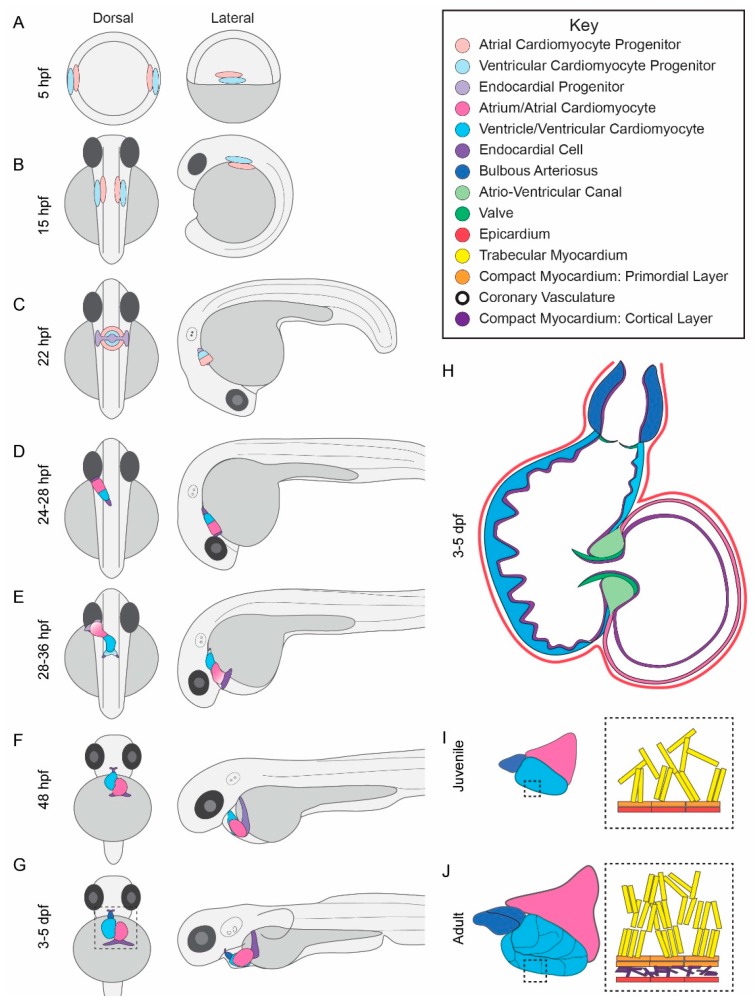
Zebrafish heart development. (**A**–**G**) Lateral and dorsal views of heart development from 5 hpf embryos to 5 days post fertilization (dpf) larvae. (**A**) Cardiac progenitors are located at the lateral margin with the ventricular progenitors more closer to the margin than the atrial progenitors at 5 hpf; **(B**) Cardiac progenitors migrate bilaterally to the anterior lateral plate mesoderm by 15 hpf; (**C**) By 22 hpf, cardiac progenitors and developing endocardial cells have fused to form the cardiac disk which begins regular contractions between 22 and 24 hpf; (**D**) From 24 to 28 hpf, the disk elongates into the linear heart tube and begins leftward migration; (**E**) The linear heart tube continues migrating leftward and begins looping. Concurrently, from 28 to 36 hpf, second heart field cells are added to the arterial and venous poles, illustrated by shading; (**F**) By 48 hpf, the two chambered heart has formed; (**G**) The bulbous arteriosus forms at the outflow tract; (**H**) Cross-sectional view of the heart from 3 to 5 featuring trabeculae located primarily in the outer ventricle wall, cardiac valves, and covering of the heart by the epicardium; (**I**) Between larval and juvenile stages, the atrium and ventricle rotate such that the atrium is dorsal to the ventricle. The inner topology is complex and features a spongy trabecular myocardium and outer compact myocardium called the primordial layer; (**J**) Additional features of the adult heart are coronary arteries which feed the ventricle and expansion of the compact myocardium by addition of a cortical layer of CMs.

When the zebrafish heart initiates contraction around 24 hpf, it is composed of three cell types—atrial cardiomyocytes (CMs), ventricular CMs, and endocardial cells [17]. These differentiated cell types can be traced to cardiac progenitor cells (CPCs), which originate at 5 hpf in the lateral marginal zone of the blastula (Figure 2A) [17,32,33,34]. The ventricular pool resides more dorsally and closer to the margin than the atrial pool, while the blastomeres that give rise to endocardial cells appear to be located across the lateral margin without any specific spatial organization [32,35,36]. These progenitors migrate during gastrulation to reside in the posterior half of anterior lateral plate mesoderm (ALPM) by 15 hpf (Figure 2B) [35,37,38,39,40]. Subsequently, these bilateral CPCs initiate differentiation programs and fuse into a disk with endocardial cells in the center lined by ventricular and atrial myocytes (Figure 2C). The disk elongates into a linear tube with distinct expression profiles for atrial CMs, ventricular CMs and endocardial cells (Figure 2D) [35,38,39,40,41,42].

The linear heart tube is originally composed of cells from the first heart field (FHF). Additional cardiac cells are recruited to the heart tube in a second wave of differentiation as late-differentiating CPC populations called the second heart field (SHF) extend the linear heart at its arterial and venous poles starting at around 28 hpf [43,44,45,46]. Concurrent with addition of the SHF-derived cardiac cells, the linear heart tube migrates leftward and begins looping (Figure 2E) [17,42,47,48]. By 48 hpf, the looped heart is located in the pericardial cavity and is clearly divided into a two-chambered heart by constriction of the atrio-ventricular (AV) canal (Figure 2F) [17,49]. Although, at 48 hpf, the major components of the heart have formed, the heart is still immature and lacks auxiliary cell types and additional structures that are important for function as the organism grows [50]. These structures include the bulbous arteriosus, valve cushions and leaflets, myocardial protrusions called trabeculae and epicardium (Figure 2G–H). These are discussed in detail, below.

*Cardiac outflow tract:* The zebrafish outflow tract is composed of the bulbous arteriosus and aorta. The bulbous arteriosus is analogous to the mammalian conotruncus and is composed of an inner layer of endothelial cells lined by a thick layer of smooth muscle cells (Figure 2H). This pseudo-chamber serves as a resistor to regulate flow through the aorta, which delivers blood directly to the gills for oxygenation [51].

*Epicardium:* The epicardium develops from an extra-cardiac population of cells called the pro-epicardium. The pro-epicardium can be distinguished morphologically at 48 hpf as a group of spherical cells located in close proximity to the ventral wall of the looped heart at the level of AV junction [52,53,54,55]. At approximately 72 hpf, the pro-epicardium expands and starts to spreads over the myocardial surface to form the epicardium (Figure 2H) [56,57]. The epicardium is an important source of signals to the underlying myocardium [58]. It also is a source of epicardial-derived, cardiac-resident cells such as cardiac fibroblasts [59,60].

*Trabeculation:* Cardiac trabeculae are highly organized, luminal, muscular ridges lined by endocardial cells in the ventricular lumen. Trabeculae increase myocardial surface area for blood oxygenation and are critical for cardiac function [27,28,53,61]. Following cardiac looping and chamber ballooning, CMs delaminate from the ventricle wall to initiate cardiac trabecular formation, and the ventricular has obvious, stereotyped trabecular ridges by 72 hpf [53,62,63]. The trabecular myocardium rapidly expands in the developing heart and as the cardiac wall matures, the trabeculae undergo extensive remodeling in association with compact myocardial proliferation, formation of the coronary vasculature and maturation of the conduction system [27]. Remodeling, also known as consolidation or compaction, marks the final stage of trabecular growth such that species-specific differences in adult trabecular morphology are generally attributed to differences in remodeling [28].

*Valvulogenesis:* Cardiac valves are a critical component of the vertebrate heart. Valves function to ensure unidirectional blood flow and prevent retrograde flow. Valve malformation underlies many forms of human congenital and adult-onset heart diseases, such as aortic or pulmonary valve stenosis, bicuspid aortic valve, mitral valve prolapse, and Epstein’s anomaly [3,49,50]. The AV canal forms at the border between the atrium and ventricle and is readily detectable during looping morphogenesis. Around 40 hpf, AV CMs expand their luminal surface while constricting their abluminal surface [49]. The underlying AV endocardial cells undergo an epithelial-to-mesenchymal transition to form the endocardial cushion, which subsequently remodels to create primitive valve leaflets allowing for complete block of retrograde blood flow at 76 hpf [49,64,65]. These leaflets continue to thicken and lengthen to form the mature valve [50].

*Late maturation:* In zebrafish, cardiac chamber maturation continues through juvenile and early adult life stages. During larval and early juvenile stages, the ventricle remodels from a grossly pyramidal shape to a more-rectangular morphology and the heart rotates such that the ventricle is positioned ventrally to the atrium (Figure 2I) [66]. In cross section, the myocardium of the ventricle wall is composed of a compact layer myocardium called the primordial layer and a spongy trabecular layer (Figure 2I) [67]. In late juvenile development leading into adulthood, the ventricle becomes more rounded and coronary arteries form at the subepicardial space to vascularize the underlying myocardium [66,68]. Additionally, a small population of inner trabecular cells breaks through the primordial layer and rapidly expands on the surface of the myocardium to form the cortical layer [67,69]. In cross section, the ventricle wall comprises two layers, a compact myocardium composed of cortical and primordial cells and the inner trabecular myocardium (Figure 2J).

### 2.2. Cardiac Morphogenesis Phenotypes in Zebrafish

Cardiogenesis is a complex process that involves elaborate tissue morphogenesis and remodeling. During this process, transcription factors and signaling networks act in a concerted fashion to generate various specialized, differentiated cardiac cell types that are subsequently assembled into a functional pumping organ. Though the zebrafish heart is small and simple compared to the human heart, it is assembled using comparable cell types and functions in similar manner. The structural and functional defects that cause heart disease in people lead to defects in heart size, shape or function in the zebrafish model. A major advantage of zebrafish embryos is that these defects can be directly observed as they develop in live zebrafish embryos.

#### 2.2.1. Congenital Heart Disease Modeled in Zebrafish

CHDs in infants feature a variety of structural malformations including remodeling defects, septal defects, valve malformations, and chamber malformations [3]. Though there is wide range in severity of CHDs, major defects ultimately lead to heart failure. Since zebrafish can meet their oxygen needs by diffusion alone during the first week of life, they can be used to study the progression of CHDs. However, due to differences in size and anatomy, heart disease in zebrafish can appear grossly different than the human analog, yet is caused by the same underlying cellular deficiency. In zebrafish, heart failure features progressive reductions in contractility which can ultimately lead to defects in heart size, shape, and function [70]. Defects in CPC dynamics may appear as cardiac bifida, hypoplastic, or hyperplastic heart in zebrafish. Likewise, genes that lead to looping defects alter the relative position of the ventricle to the atrium. Defects in chamber maturation can cause hypotrabeculation, and impaired valvulogenesis leads to altered intracardiac fluid dynamics. Similarly, improper development or functional perturbation of the conduction system appears in zebrafish as arrhythmias, which can be directly observed or assessed using genetically encoded calcium sensors [70].

#### 2.2.2. Genes That Cause CHD in Humans Cause Heart Malformation in Zebrafish

Both genetic and nongenetic factors can cause CHD. As reviewed by the Seidman group, there is remarkable genetic heterogeneity in CHD [6]. CHD mutations found in infants impact a wide range of molecules and usually alter the effective dose of a gene product. However, identical mutations can lead to different cardiac malformations, and identical malformations may be caused by different mutations. Occasionally, the genetics of CHD are more straightforward such as the situation in which single mutations cause rare, familial CHD. As described in Table 1, genes encoding key transcription factors, cell signaling molecules, and contractile machinery have been shown to cause CHD in humans and are important for heart development in zebrafish.

#### 2.2.3. Non-Genetic Factors That Cause CHD in Humans Cause Heart Malformation in Zebrafish

Non-genetic factors can modulate heart morphogenesis by perturbing gene networks to alter cell function. Two major non-genetic factors are well known to play a role in this manner—hemodynamic forces and cardiotoxic agents.

The heart develops concurrent with heartbeat, and the biomechanical forces created by heartbeat influence cardiac morphogenesis [71,72]. In chick and zebrafish models, mechanical disruption of normal blood flow leads to major cardiac malformations [73,74,75]. Genes that are involved in the production or detection of hemodynamic force are implicated in human heart disease (Table 1). The mechanisms by which mechanical forces regulate cardiac morphogenesis are poorly understood, but are thought to involve coordinated cellular responses to shear stress (the frictional force of flow), stretch, and chamber pressure. In zebrafish, trabeculation is highly sensitive to flow patterning [62,63,76]. Additionally, there is a tight interrelationship between flow magnitude and directionality and valvulogenesis [77].

Pharmacological, environmental, and infectious agents are well known to cause cardiotoxicity in people and are the focus of several excellent reviews [78,79]. Some agents, such as ion or calcium inhibitors, impair cardiac function [80]. Others, such as alcohol or thalidomide, lead to congenital malformations [81,82]. It is important to note that the developing fish heart is a sensitive target organ, and several environmental and anthropogenic toxicants are capable of causing cardiotoxicity in zebrafish [83,84,85,86,87]. Thus, zebrafish can be used as a good predictive model for their toxic capacity [79,88,89].

#### 2.2.4. Cardiomyopathy Modeled in Zebrafish

Cardiomyopathy refers to any disease of the heart muscle. Structural malformations found in congenital heart diseases can lead to cardiomyopathy as CMs undergo pathological remodeling to maintain cardiac output with suboptimal chamber structure. This remodeling, though initially compensatory, is ultimately unsustainable and leads to heart failure. Similarly, cardiomyopathies can also be caused by deficiencies in CM contractile machinery whereby CMs lack the ability to produce sufficient contractile force. This leads to hypertrophic or dilated cardiomyopathy and eventual heart failure [90]. Zebrafish that carry mutations in sarcomere genes implicated in human heart disease have contractility defects, which ultimately lead to chamber collapse—the zebrafish equivalent to heart failure (Table 1) [91,92]. In addition to functioning contractile machinery, coordinated contraction requires proper expression and localization of cardiac ion channels that produce and regulate the cardiac action potential. The mechanisms underlying myocardial automaticity, refractoriness and conduction are highly conserved between humans and zebrafish [93]. Defects in ion channels can lead to fatal arrhythmias in humans. Since zebrafish embryos can survive for the first week of life with major cardiovascular defects, arrhythmias can be directly observed [80]. Zebrafish carrying mutations in genes important for the human cardiac action potential display as tachycardia, bradycardia, and/or or alterations to the CM calcium wave (Table 1).

## 3. Experimental Approaches to Studying CHDs in Zebrafish

**Figure 3:**
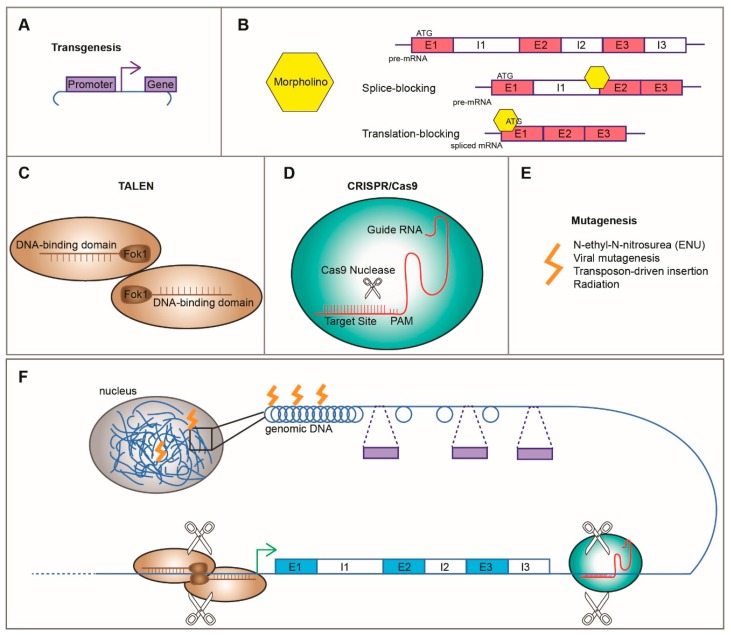
Common tools used in zebrafish include (**A**) transgenic insertion of foreign DNA; (**B**) suppression of gene expression by splice-blocking or translation-blocking morpholinos; (**C**) targeted genome editing by TALE-associated nucleases (TALEN); (**D**) targeted genome editing by CRISPR-associated Cas9 nucleases; and (**E**) chemical mutagenesis; (**F**) Schematic illustrating how these tools modify genomic DNA.

Though the molecular basis of many familial heart diseases is known, the specific causes of most heart diseases are still poorly understood [94,95]. Due to the high rate of genetic conservation to humans and their amenability to genetic manipulation, zebrafish are well suited for studying the molecular basis of cardiovascular disease [96,97,98,99]. Fortunately, in addition to phenotype-based genetic mutagenesis screens, zebrafish are highly amenable to newly developed genome editing approaches that facilitate functional characterization of candidate genes, visualization of cardiac structures, and analysis of cell signaling [100,101]. The major molecular and genetic tools used in zebrafish genetics are discussed below and summarized in Figure 3.

Zebrafish are used to model human heart disease through hypothesis-driven reverse approaches (functional characterization of candidate genes and cardiotoxicity models) [102,103] and data-driven, forward approaches (random mutagenesis, forward genetic screens, high throughput small molecule screens) [104], which contribute to understanding the etiology, phenotype, and treatment of heart disease. Here, we discuss some of the ways in which the zebrafish are used to produce important insights into heart development and disease.

### 3.1. Hypothesis Driven/Reverse Genetics

By current estimate, there is approximately 82% conservation between zebrafish and humans for disease-related genes [105]. The zebrafish genome is sequenced and has undergone ten annotations with the upcoming annotation to be completed by 2017 [14]. However, with over 26,000 genes in the genome, understanding the specific role of individual genes in heart development and disease is a massive undertaking [99]. To add to the complexity, there was a full genome duplication in teleost fish that has led to functional duplication or divergence for some genes [106]. In the post-genomic era, complete sequencing of the zebrafish genome and development of tools for gene knockdown and targeted genome modification has enabled functional characterization nearly any candidate gene [107]. Here, we summarize tools and reverse approaches for hypothesis-driven study of heart development and disease in zebrafish.

#### 3.1.1. Transgenesis

*Transgenesis*—the stable integration of foreign DNA into the genome—is a critical part of the zebrafish toolbox (Figure 3B). Through manipulation the transgenic construct, stable genetically engineered zebrafish can be made that produce proteins for a variety of purposes. Transgenic zebrafish lines have been generated to temporally and spatially overexpress genes of interest, to label tissues or specific cell types with fluorophores, and to report activation of cell signaling pathways [18,19,20]. The myriad functional applications for transgenics are outlined in Box 1.

Transgenic zebrafish are also used to overexpress defective proteins, which are predicted to be causative in human cardiac diseases [108,109]. This is particularly effective when the mutated (or affected) protein is predicted to have dominant effects. For example, Huttner *et al.* created a transgenic zebrafish line that stably expresses a human arrhythmogenic *SCNA5A* channel variant and consequently displays bradycardia and conduction abnormalities [109]. This approach of expressing a human variant in zebrafish again underlines that the zebrafish is a useful *in vivo* tool to evaluate human disease-associated gene variants in cardiac ion channel genes in a time and cost efficient manner.

**Box 1: Transgenic zebrafish applications**

In transgenic zebrafish, a variety of methods can be used to stably integrate an engineered piece of DNA randomly into the genome [110]. The transgene typically consists of a ubiquitous or cell-type specific promoter sequence that drives expression of a gene or gene variant of interest. By varying the promoter and gene, transgenic zebrafish can be engineered for a variety of functions, some of which are described below. It is important to note that it is possible for random integration of the transgene to disrupt endogenous gene expression in a deleterious manner. To account for this possibility, it is standard practice to generat multiple stable lines for each transgenic and retain only those lines with comparable phenotypes.

(1) Spatial control of gene expression is achieved by engineering transgenes to have cell type specific promoter sequences driving gene expression. By varying the gene expressed, transgenes can label subcellular features, trace cell lineages, or even selectively kill a specific cell type. For example, the promoter for the *cardiac myosin light chain 2* (aka, *myl7*), is expressed exclusively in CMs. Thus, CMs in *Tg*(*cmlc2:GFP*) zebrafish express the green fluorescent protein, GFP [111]. *Tg(cmlc2:dsRed)* zebrafish express red fluorescent protein dsRed [112]. Similarly, *Tg(cmlc2:DTA)* zebrafish express DTA (diphtheria toxin A) to selectively kill CMs [113]. Likewise, *Tg(cmlc2:Cre-ER^T2^)* CMs express the fusion protein of Cre recombinase and ER^T2^ [114], which is a tamoxifen-inducible portion of the estrogen receptor. In the presence of tamoxifen, Cre-ER^T2^ translocates to the nuclease where it recognizes and recombines loxP sites. The Cre-Lox system can be used for gene deletion or lineage tracing.
(2) Temporal control of gene expression has been achieved using heat-shock protein promoters. For example, in *Tg(Hsp70:sema3aa)* zebrafish [115], when the organism is exposed to elevated temperature, Heat Shock Factor binds to the *Hsp70* promoter to transiently activate transcription of the downstream gene *sema3aa*.
(3) Overexpression: The functional consequences of over-activation of the gene of interest can be assessed by designing a transgenic construct where the promoter drives expression of the gene of interest. Expression level can be titrated by altering transgene copy number and promoter strength. This is particularly useful if the gene of interest has dominant activity.
(4) Biosensors: Transgenic reporter lines can be made to express biosensors under the control of signaling pathway-responsive elements, making useful and reliable molecular tools to decipher the activation or inhibition of distinct cell signaling events. These biosensors are additionally, valuable resources for drug screening. For example, in *Tg*(*tp1:EGFP*) zebrafish, the Notch response element tp1 is upstream of the fluorescent protein EGFP such that cells with active Notch signaling express EGFP [116].

#### 3.1.2. Morpholinos

Targeted knockdown of genes in zebrafish has been efficiently achieved by injection of anti-sense morpholinos (MOs) (Figure 3E). MOs are synthetically modified, oligonucleotides designed to bind to either translation-initiation site or splicing donor/acceptor sites of mRNAs or pre-mRNAs, respectively. MOs are delivered by microinjection into embryos at the 1–4 cell stage where they transiently knock down gene function post-transcriptionally and are slowly degraded by normal cellular processes [117,118,119]. For this reason, MOs are only effective for knocking down gene expression in embryos. MOs have been widely used to assess the effect of knocking down gene expression quickly and inexpensively relative to generating gene-targeted mutants [87,120,121] and they have been adapted for use in other fish models including *Fundulus heteroclitus* [122,123].

However, there are certain limitations to and disadvantages of this technique. While some morphants recapitulate a genetic mutant phenotype, others do not. It has been proposed that the discrepancy between mutant and morphant phenotypes is likely due to off-target or even non-specific effects of the MO [124,125]. Recent evidence suggests that it could also be due to genetic compensation to counterbalance deleterious mutations through mechanisms activated in mutants but not morphants [126]. Therefore, targeted gene inactivation experiments via MO in zebrafish should be carefully conducted and well-controlled as described in Box 2.

Additional applications for MOs include the systematic use of morpholino-mediated knock-down in zebrafish embryos carrying deleterious mutations or treated with cardiotoxic agents to identify disease-suppressing genes and novel drug targets [86,87].

**Box 2: Morpholino Controls**

Morpholino oligonucleotides (MO) recognize ~25 base pair sequences of RNA and interfere with gene translation or splicing [127]. Like other sequence-based tools for gene knock down can have off-target effects derived from binding to other occurrences of the 25 base pair sequence, toleration of some degree of mismatch, and/or toxicity associated with high concentrations of MO in the cell. Recent work comparing mutant and morphant phenotypes have called into question the specificity of MOs and subsequently their utility in biological research [125]. However, carefully controlled MO experiments can limit potential off-target effects of MOs, and so long as appropriate controls are used, MOs can be an important first tool for assessing gene function [128]. We direct the interested reader to Eisen and Smith [118] and Bill *et al.* [119] for a complete description of accepted standards for morpholino experiments, summarized below:

Efficacy:
  (1) Dose-dependent reduction in endogenous protein
  (2) Dose-dependent reduction in properly spliced transcript (splice-blocking MO only)
  (3) Dose-dependent reduction in tagged version of target protein
Specificity:
  (1) MO sequence selection (BLAST)
  (2) Comparison to existing mutant homozygous for null allele
  (3) Co-injection with *in vitro* transcribed target RNA
  (4) Control morpholino (standard control, 5-base pair mismatch, and p53 MOs)
  (5) Multiple MOs targeting the same gene lead to similar, synergistic phenotypes

#### 3.1.3. Genome Editing

Genome editing provides the opportunity to bring virtually every mutation found in diseased humans into the zebrafish genome, thereby enabling evaluation of the impact of small molecules on the disease phenotype caused by specific mutations. One of the advantages of zebrafish as a model organism is the availability of engineered endonucleases including zinc finger nucleases (ZFNs), transcription activator-like effector nucleases (TALENs), and clustered regularly interspaced short palindromic repeat (CRISPR) RNA-guided Cas9 nucleases [108,129]. TALENs and CRISPR/Cas9 in particular, are very efficient for making targeted lesions in the zebrafish genome. With these tools, in a relatively short time span, endogenous genes can be targeted to generate loss-of-function alleles. Additionally, endogenous genes can be replaced with “knock-in” reporter constructs [108,130]. Though the mechanism of action for each tool is different, these modifications are made when the engineered nuclease binds specific DNA sequences and causes double strand breaks (DSBs) which are repaired through non-homologous end joining or homologous template dependent repair, ultimately leading to indel mutations or DNA replacement [102]. These modifications can be transmitted to the germline and passed on to future generations [131]. Genome editing both facilitates conventional drug discovery and represents a significant milestone toward the future of personalized medicine. The aforementioned molecular tools are reviewed in detail below.

ZFNs were the first available direct site-specific gene targeting tool used for zebrafish genome editing. These chimeric proteins contain a DNA binding domain comprised of a C2H2 type zinc finger array (ZFA) and a cleavage domain derived from Fok I, a bacterial non-specific endonuclease. The helix domain of each ZF recognizes and binds to specific, 3 bp DNA sequences [132]. ZFN technology has been successfully applied in zebrafish to knock out *gata2a* resulting in circulatory defects in mutant embryos. Selection of ZFAs with high specificity and efficiency of DNA binding activity remains a challenge and is limiting [129]. Additionally, commercial service for ZFN generation and screening is more expensive than TALEN and CRISPR/Cas9 nuclease engineering, making this genome editing technique less ideal than other options.

TAL effector nucleases (TALENs) were originally derived from Xanthomonas bacteria. TALENs are fusions of the FokI cleavage domain and DNA-binding domains derived from TALE proteins. TALEs contain multiple 33–35-amino-acid repeat domains that each recognizes a single base pair. Typically, target sties of 12 bp or longer are necessary for TALEN specificity; however, it can be difficult to construct long TALE repeats because of their highly repetitive sequence [103,133]. TALENs have been broadly applied in zebrafish, and recent *egfl7* mutant showed embryonic cardiovascular defects [126].

The bacterial and archaeal adaptive defense mechanism CRISPR (clustered regularly interspaced short palindromic repeats) is the newest and most nimble addition to the zebrafish gene-editing toolbox. Type II CRISPR systems are especially useful as reverse genetic tools because they require only the enzyme Cas9 and a single guide RNA (sgRNA) which is engineered through standard DNA cloning techniques, to recognize a target site of 20 nucleotides followed by NGG [108,134]. Since this sequence occurs frequently in the genome, sgRNAs can be designed to target virtually every gene in the genome. However, with only 20 bp for sequence specificity and some promiscuity of sgRNA binding, this approach is prone to off-target effects [135]. Current estimates suggest that the rates of mutagenesis at potential off-target sites with CRISPR are low (1%–3%) [136,137,138]. Box 3 discusses common approaches for mitigating potential off-target effects in zebrafish genome editing. CRISPR/Cas9 has been engineered to efficiently mutate specific loci in zebrafish both to generate mosaic individuals that harbor a range of mutations and to generate stable lines carrying a single modified allele [102].

**Box 3: Limiting CRISPR/Cas9 off-target effects**

Though the CRISPR/Cas9 system is a convenient and highly efficient tool for gene editing in zebrafish, as a sequence-based technology it is vulnerable to off-target effects [139]. Recent technical advances have improved technologies for detecting and quantifying off-target cleavage [140]. However, these technologies are often prohibitively expensive for large scale use. While simple steps can be taken to mitigate off-target effects in CRISPR/Cas9 genome editing (described below), this is an area of very active research and we direct the interested reader to a recent review [141].

(1) Strategic gRNA design—Use computational methods to predict guide RNA specificity to the target site [142].
(2) Cas9 modifications—Versions of Cas9 are available that require two guide RNAs for nuclease activity, decreasing the probability of double stranded breaks at unintended sites. These include Cas9 nickase [143] and Cas9-Fok1 nuclease fusion proteins [144].
(3) For each desired genome editing process, generate at least 2 independent lines using different gRNAs.
(4) Outcross to wild type to isolate desired mutation from non-linked off-target mutations.

## 4. Adult Functional Assays

Since selective pressures in controlled laboratory conditions are limited, zebrafish can survive with phenotypic defects in cardiorespiratory capacity that would be undetectable without external stressors [145,146,147,148]. Thus, genetic defects that lead to mild reductions in cardiovascular function might be clinically relevant, but not readily observed in the embryonic zebrafish heart.

In mammals, cardiac functional parameters such as fractional shortening, heart rate, cardiac output and end systolic and end diastolic volumes are measured via echocardiography (ECHO) [149,150]. By combining conventional echocardiography with modern speckle-tracking analyses, a recent study has overcome the challenge of small heart and body size of the adult zebrafish to perform highly sensitive non-invasive assessment of cardiac performance [151]. Alternatively, heart function can be inferred from measures of total cardiorespiratory performance. Cardiorespiratory performance is evaluated in rodent models via exercise testing, which provides information about cardiovascular health by assessing maximal exercise endurance and/or maximum oxygen consumption (VO2 max), as extensively reviewed in Marcaletti *et al.* [152]. VO2 max is a highly reproducible apical endpoint defining the physiological capacity of an individual’s cardiovascular system [152].

Swimming capacity and respiratory function are central determinants of fish fitness [153,154,155]. The most widely used measurement of swimming performance in fish is critical swimming speed (Ucrit)—the maximum velocity a fish can maintain throughout a prolonged swimming period using a swim tunnel respirometer [156,157]. Maximum aerobic performance during Ucrit utilizes the maximum pumping capacity of the heart, providing a physiological measurement of cardiovascular health and function [158,159,160]. Use of Ucrit as a cardiorespiratory gauge in larval or adult zebrafish has demonstrated that altered cardiovascular morphology/function impacts swimming performance in the face of various stressors including temperature, hypoxia, exercise, and contaminant exposure [161,162,163].

## 5. Data Driven/Forward Approach

The small size, transparent embryos, short generation time, and high fecundity make zebrafish more amenable to high-throughput, data driven applications such as forward genetics and drug screens. These unique attributes also make them ideal candidates for robust statistics in screening approaches. The first large-scale zebrafish forward genetic screens provided the basis for the discovery of numerous novel genetic players critical for vertebrate development [14,15,17,99,164,165]. Following is an overview of the major applications of data driven approaches, focusing on forward genetic screens and high throughput methodology.

### 5.1. Mutagenesis

Several strategies have been developed to mutagenize the zebrafish genome, including *N-*ethyl-*N*-nitrosourea (ENU) based chemical mutagenesis, viral and transposon-driven insertional mutagenesis (Figure 3E) [166,167,168]. The first, large scale, vertebrate ENU-based mutagenesis screens were conducted in zebrafish and this screen has provided novel insight into the genetic regulation of early vertebrate development [169]. Zebrafish mutants are often named according to the phenotype of the mutation, e.g., *silent heart* mutants have a heart that does not contract. The causative mutation can be identified through positional cloning or next generation sequencing [170,171,172]. After the causative mutation is identified, the mutant allele is referred to by the gene symbol.

#### 5.1.1. Forward Chemical Genetic Screens

Since the first genetic screens in 1996, hundreds of mutant zebrafish lines have been identified through large-scale forward mutagenesis screens [25,104,173]. Much of what we know about the molecular basis of heart development has come from characterizing these mutants. Typically, in forward genetic screens, mutagenized males are crossed to wildtype females and the resulting F1 offspring are raised to adulthood. After outcrossing of the F1 adults to produce an F2 generation, cardiovascular phenotypes are screened in F3 embryos from sibling crosses within the F2 families. Given that the heart is the first organ to function, and its formation is well characterized, cardiovascular phenotypes are relatively easy to identify in these offspring. Zebrafish can also be used for non-conventional forward genetic screens, including non-complementation, deletion, modifier, and sensitized screens [104]. The function of many genes identified in forward screens causing cardiovascular phenotypes in zebrafish are also perturbed in human heart disease, which demonstrates the conservation of gene function between zebrafish and human [104,170].

#### 5.1.2. Zebrafish in High Throughput Chemical Screens

Small chemical compound screens have been the standard method for pharmaceutical drug discovery for decades. Low success rates in drug discovery—only 3% of newly designed drugs against novel targets reach preclinical studies—demonstrated the need for large-scale high throughput screens [174] platforms for the efficient and beneficial screening of thousands of small bioactive molecules per day to identify novel treatment strategies [10,11,175,176,177]. Genomic knowledge and research, synthetic chemistry, and computerized laboratory robotic automation have all helped to accelerate the identification of cardiovascular disease therapeutics in whole-organism-based systems.

High throughput tools and technologies for working with zebrafish are evolving rapidly and creating new opportunities to contribute to improved drug development and personalized patient treatment options. During the last decade, drug screens performed in zebrafish have evolved from manual and semi-automated systems partly requiring individual placement of embryos or manual data acquisition to fully automated high throughput screens (HTS) [178,179]. Robotics facilitates embryo dispensation, compound delivery, and incubation, as well as image acquisition and analysis of a variety of parameters to grasp the complexity of cardiac function [9,180,181].

Burns *et al.* employed the transgenic line *Tg(cmlc2:GFP)* that expresses green fluorescent protein (GFP) exclusively in the myocardium to assess the heart rate of the embryos under the influence of antiarrhythmic drugs by digital imaging [177]. Similarly, Yozzo *et al.* established a 384-well-based high-throughput assay using transgenic *Tg(fli1:EGFP)* zebrafish embryos that express EGFP in vascular endothelial cells. This transgene allows for automated quantification of heart rate, blood circulation, body length, and intersegmental vessel formation [11].

The parameters that can be addressed by HTS continue to expand. Lin *et al.* recently developed a pseudodynamic three-dimensional imaging system that measures heart rate, ventricular stroke volume, ejection fraction, cardiac output, diastolic filling, and ventricular mass larvae up to 6 dpf [78]. However, automated HTS assays do not necessarily require transgenic reporter lines. Additionally, Letamendia *et al.* developed a fully-automated screening platform that dispenses embryos onto 96-well plates, administers the chemical compounds, incubates the embryos with the compound, and acquires images for phenotypic analysis [176].

Zebrafish are extremely valuable model for identifying therapeutics that can rescue congenital heart disease phenotypes. Peterson *et al.* performed one of the first successful whole-organism-based, phenotype-guided therapeutic small compound screens using the zebrafish mutant line *gridlock* [182]. *gridlock* features a mutation of the *hairy/enhancer of split-related with YRPW motif 2* (*hey2*) gene, and homozygous *gridlock* mutants display a phenotype analogous to coarctation of the aorta in humans [183]. Following a 5000 compound screen, two structurally related compounds were identified to up-regulate VEGF and suppress the coarctation phenotype in *gridlock* mutants *in vivo* [182].

Additionally, zebrafish have been used to identify small molecules that can rescue arrhythmia defects. The zebrafish *breakdance* mutants exhibit prolonged cardiac repolarization and QT prolongation due to a loss of function mutation of *potassium voltage-gated channel*, *subfamily H (eag-related)*, *member 2a* (*kcnh2*). The zebrafish *reggae* mutation is a gain-of-function mutation of *potassium voltage-gated channel*, *subfamily H (eag-related)*, *member 6a* (*kcnh6a*) and results in a short QT phenotype [184]. In a small compound screen (SCS) with 1200 compounds, Peal *et al.* identified two drugs that rescued *kcnh2* mutant long-QT phenotype [185].

#### 5.1.3. Cardiotoxicity Screens

The understanding of molecular interactions, off-target, and toxic effects is an essential part of the drug discovery process. *In vitro* assays are appropriate for evaluating cytotoxicity, but not for assessing pharmaceutical efficacy in the context of complex, multi-organ interactions. The zebrafish complements well-established *in vitro* assays and traditional preclinical *in vivo* models [9,78,179]. One of the most important types of drug toxicity is cardiotoxicity, which is often characterized by impaired cardiac contractility or arrhythmias [186,187,188]. Consequently, the zebrafish has emerged as a great model system to assess therapeutics for modulating contractility defects and arrhythmias. Zebrafish are used both to assess the ability of a therapeutic agent to induce off-target cardiotoxic effects and alleviate symptoms of cardiotoxicity. Furthermore, cellular and molecular responses to small molecules are well conserved between humans and zebrafish [7,105]. For these reasons, the zebrafish has been widely used in toxicological studies investigating the effects of environmental contaminants on heart development and function [88,89,189].

Given that zebrafish experience similar drug induced cardiotoxicity, the model serves as a great system for therapeutic trials/screens. Drugs known to have cardiotoxic effects clinically, including Doxorubicin, Sorafenib, Sunitinib, and aristolochic acid recapitulate cardiotoxicity in zebrafish embryos [190,191]. Additionally, human cardiomyopathy-associated proteins, such as amyloid light-chain, induce cardiac dysfunction and death when injected into zebrafish embryo [192]. Doxorubicin induces cardiomyopathy and heart failure within four days of treatment and embryos treated with aristolochic acid develop cardiac hypertrophy and acute progression of heart failure within two days. These symptoms can be attenuated by administration β-blockers and ACE-inhibitors known to attenuate heart failure as well as small molecules identified from chemical library screening of Doxorubicin-treated embryos [79]. Similarly, zebrafish have been used to model drug-induced QT-prolongation where an initial screen determined that drugs known for their QT-prolonging effect in humans consistently triggered bradycardia and AV-block by interfering with repolarization and cardiac conduction [193].

## 6. Future Directions

The rise of next generation sequencing and genome editing technologies, coupled with the unique advantages of the zebrafish model, may lead to novel insight into the etiology of CHDs. However, the zebrafish model continues to evolve as it is more frequently used as a complementary animal model system to model and study the underlying etiology of human heart diseases. Specific areas of improvement include characterization of cardiac disease phenotypes and developing tools to assess cardiotoxicity in adult zebrafish. As technologies of disease modeling and small compound screening in zebrafish improve further, efficiency of targeted drug discovery using zebrafish will increase.

### 6.1. Characterizing Cardiac Phenotypes in Embryos

There is still a need to rapidly characterize cardiac disease phenotypes. Early studies of cardiac development relied heavily on the analysis of static cardiac and embryonic samples [173]. Yet, these approaches failed to capture the dynamic process of cardiac development. The strength of the zebrafish model system lies in the transparent embryo that offers the possibility of live dynamic imaging and analysis of developmental cardiac defects at both cellular and subcellular resolution using cardiac-specific fluorescent transgenes and high-speed fluorescent microscopy. Such detailed cellular and subcellular studies will likely offer a better understanding of the dynamics of each cardiac cell, cell signaling events, and the cellular response to these signals with high temporal and spatial resolution. Characterizing these developmental benchmarks will provide greater mechanistic insight into cardiac development and CHDs.

### 6.2. Adult Zebrafish Disease Model

The majority of zebrafish studies are conducted on the embryos because of their advantages such as transparency, small size, and limited space requirements to achieve highest throughput. Unfortunately, current studies using embryonic zebrafish can only complement mammalian studies, and the findings might not necessarily be translatable to diseased adult humans, in which comorbidities are common and environmental stressors are present. The adult zebrafish is a feasible option for a variety of studies and drug screens, and has proven to be an economic alternative to other animal models. Consequently, adult zebrafish should be used to investigate later-life cardiac disease states. Studies have shown that adult zebrafish can be used to examine later-life behavior and cardiac regeneration in addition to cardiotoxic evaluation, and adult zebrafish have also been recently used to model adult cardiomyopathy [194].

### 6.3. Personalized Medicine

CRISPR/Cas9 genome editing in combination with the power of the zebrafish platform holds great promise for personalized medicine. Should sequencing of the full exome of a patient with familial heart diseases reveal the potential causative mutations, CRISPR/Cas9 genome editing can be used to introduce mutations in the zebrafish genome to mimic human disease gene variants to assess the causal effects on human disease phenotype. High throughput screening techniques can be subsequently applied to the patient-specific zebrafish model to help identify effective therapeutics.

## 7. Conclusions

Over the last two decades, zebrafish have emerged as a premier model organism for exploring the molecular underpinnings of heart development and disease. The zebrafish heart develops rapidly in a stereotyped manner that largely recapitulates early mammalian cardiac development. Leveraging the vast capacity of this model system, forward genetic screens have led to important insights into cardiac morphogenesis. Recently, genome-editing techniques offer the potential to characterize candidate genes and progress in optimizing high throughput screening of zebrafish embryos has opened the doors for zebrafish as a tool for personalized medicine.

## Figures and Tables

**Table 1:** List of human congenital heart diseases (CHD) genes and their zebrafish orthologues.

Gene	Gene name	Human CHD	Zebrafish	Pubmed ID
**Transcription Factors**				
*GATA4/5/6*	GATA4 transcription factor	Septal defects, valve malformation, Tetrology of Fallot	CM specification	12845333, 24638895, 23289003, 24841381, 16079152, 10580005, 17950269, 17869240
*NKX2.5*	Homeobox containing transcription factor 2-5	Septal defects, conduction abnormalities	Looping, CM proliferation, CM differentiation	9651244, 19158954
*TBX5*	T-box 5 transcription factor	Septal defects and Tetrology of Fallot in Holt-Oram Syndrome	Bradycardia, looping	8988164, 12223419
*HAND2*	Helix-loop-helix transcription factor	Tetrology of Fallot	Cardiac differentiation	26676105, 17681136, 10821756
**Cell Signaling and Growth Factors**				
*NOTCH1*	Notch homolog1	Valve malformation, outflow tract	Valvulogenesis, conduction tissue specification, trabeculation	18593716, 16025100, 16481353, 14701881, 26628092
*SMAD6*	SMAD family member 6	Septal defects, valve malformation, coractation of the aorta	CM proliferation	22275001, 22247485
*FLK1*	Vascular endothelial growth factor	Coractation of the aorta, outflow tract defects	Valvulogenesis	20420808, 16170785
*SEMA3*	Semaphorin 3	Anomalous pulmonary vein connection	Primary heart field size	23685842, 16860789
**Cardiomyocyte Function**				
*MYH6*	Alpha myosin heavy chain	Atrial septal defect, left ventricular non-compaction, cardiomyopathy	Atrial contraction	15735645, 17611253, 14573521
*ACTC*	Alpha cardiac actin	Atrial septal defect	Endocardial cushion morphogenesis	17947298, 22751927
*TITIN*	Titin	Cardiomyopathy	Sarcomere assembly	22335739, 11788825, 9007227
*KCNH2*	Potassium channel, voltage gated eag related subfamily H, member 2	Long QT syndrome, short QT syndrome, atrial fibrillation	Ventricular asystole, QT intreval	15828882, 19668779, 17592134, 18250272, 14678746
